# Advancing diagnostics and disease modeling: current concepts in biofabrication of soft microfluidic systems

**DOI:** 10.1007/s44164-024-00072-5

**Published:** 2024-06-04

**Authors:** César R. Casanova, Marta R. Casanova, Rui L. Reis, Joaquim M. Oliveira

**Affiliations:** 1 3B’s Research Group, European Institute of Excellence in Tissue Engineering and Regenerative Medicine Headquarters, Parque de Ciência e Tecnologia, I3Bs – Research Institute on Biomaterials, Biodegradable and Biomimetics - University of Minho, Zona Industrial da Gandra - Avepark, Barco, Guimaraes, 4805-017 Portugal; 2 ICVS/3B’s – PT Government Associate Laboratory, Guimaraes, Braga, Portugal

**Keywords:** Biofabrication, Diagnostic chip, Disease modeling, In vitro models, Microfluidics, Organ-on-a-chip, Soft microfluidics

## Abstract

Soft microfluidic systems play a pivotal role in personalized medicine, particularly in in vitro diagnostics tools and disease modeling. These systems offer unprecedented precision and versatility, enabling the creation of intricate three-dimensional (3D) tissue models that can closely emulate both physiological and pathophysiological conditions. By leveraging innovative biomaterials and bioinks, soft microfluidic systems can circumvent the current limitations involving the use of polydimethylsiloxane (PDMS), thus facilitating the development of customizable systems capable of sustaining the functions of encapsulated cells and mimicking complex biological microenvironments. The integration of lab-on-a-chip technologies with soft nanodevices further enhances disease models, paving the way for tailored therapeutic strategies. The current research concepts underscore the transformative potential of soft microfluidic systems, exemplified by recent breakthroughs in soft lithography and 3D (bio)printing. Novel applications, such as multi-layered tissues-on-chips and skin-on-a-chip devices, demonstrate significant advancements in disease modeling and personalized medicine. However, further exploration is warranted to address challenges in replicating intricate tissue structures while ensuring scalability and reproducibility. This exploration promises to drive innovation in biomedical research and healthcare, thus offering new insights and solutions to complex medical challenges and unmet needs.

## Introduction

**Fig. 1:**
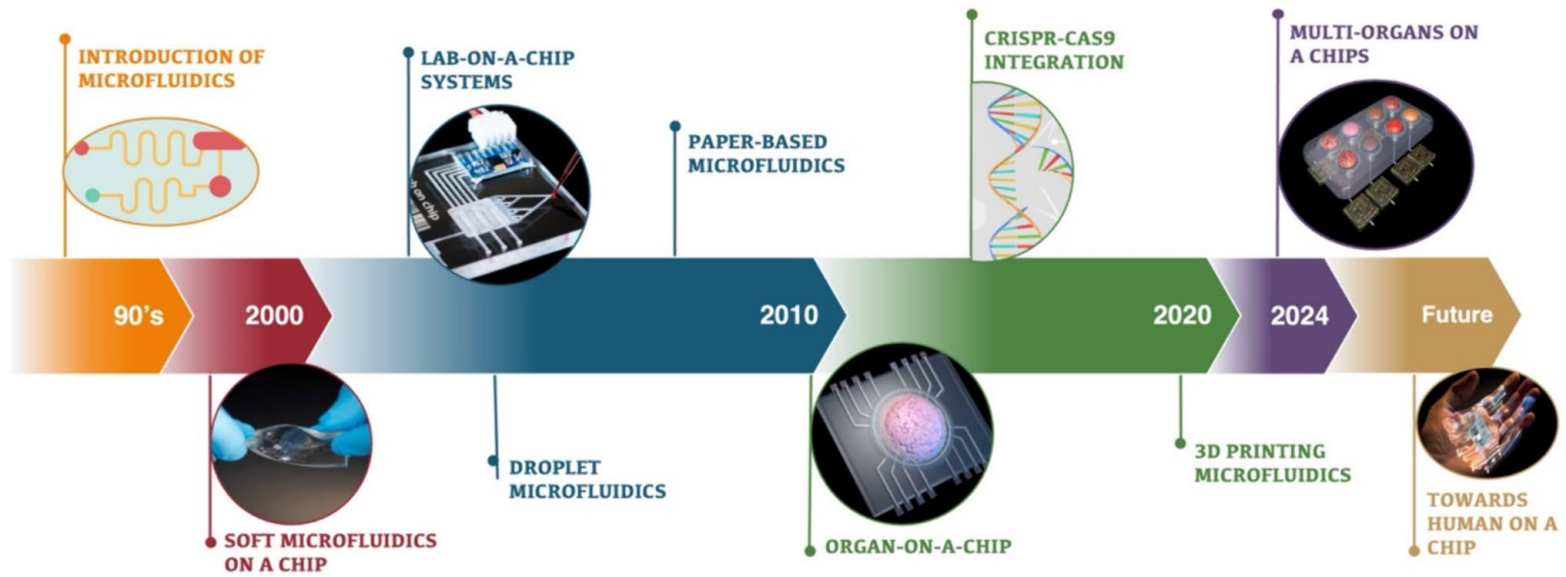
Evolution of microfluidics: from introduction to future innovations

In the dynamic landscape of biomedical engineering, the evolution of microfluidics (Fig. 1) has been instrumental in revolutionizing research methodologies and therapeutic interventions [1, 2]. Particularly noteworthy is the emergence of soft microfluidic systems, which have ushered in ground-breaking approaches to diagnostics and disease modeling, offering unprecedented precision and versatility [3, 4].

Microfluidics, characterized by manipulating fluids at a microscale, finds applications across diverse disciplines such as chemistry, biology, and medicine. However, the most significant advancements have been witnessed within the realm of soft microfluidics, owing to its ability to emulate physiological conditions more closely [5, 6].

This concept paper aims to provide an insightful exploration and concise overview on the state-of-the-art techniques utilized in biofabrication, with a special focus on soft microfluidics. By delving into the innovative biomaterials and bioinks employed, as well as the sophisticated designs implemented, the transformative potential of these technologies in diagnostics and disease modeling will be elucidated.

Central to the discussion is the recognition of the pivotal role played by three-dimensional (3D) in vitro models and bioinks in advancing biomedical research [6, 7]. These tools enable researchers to create more accurate representations of human tissues and organs, thereby enhancing understanding of tissue functioning and disease pathology, and thus facilitating the development of tailored therapeutic strategies [1, 7]. Moreover, the importance of integrating lab-on-a-chip technologies with soft nanodevices is gaining a new impetus to address the current societal challenges aiming to develop cost-effective advanced research tools and mimetic disease models to be used as alternatives to animal models. By synergizing biofabrication techniques with advanced soft materials and microfluidic platforms, researchers can create highly customizable systems capable of mimicking complex mechanical and biological environments with unparalleled fidelity [8].

## Cutting-edge concepts: exploring the frontiers of biofabrication and soft microfluidic systems

Recent breakthroughs in biofabrication technology have led to significant progress in the development of soft microfluidic systems, as demonstrated in Table 1 [9–14], detailing different techniques along with their corresponding materials and applications in disease modeling, addressing various challenges.

**Table 1:** Advances in soft microfluidic devices for health research: cutting-edge developments in diagnosis and disease modeling

Microfabrication method	Materials	Applications/disease modeling	Challenges addressed	*Main advantages*	*Main disadvantages*	Ref.
High resolution 3D printing	PEGDA-250, TPO, ITX, and PETTA	- Fabrication of microfluidic organ-on-a-chip devices and microfluidic components, capillary flow-based systems.	- Rapid polymerization under low irradiance, reliable printing of embedded microchannels and thin membranes despite illumination inhomogeneity - Low viscosity for fast printing, low protein adsorption, and cytocompatibility	*- High-resolution and rapid micro-fabrication* *- Cost-effective mass production* *- Versatility in applications* *- Automated fabrication process*	*- Limited by the resolution and size of the LCD pixels* *- Challenges with illumination inhomogeneity affecting printing quality*	[9]
3D-printed soft lithography	PDMS	- Reconstruction of complex neural circuits - Powerful platform for studying neurodegenerative diseases	- Creating complex, compartmentalized microfluidic devices with high-aspect-ratio features - Simplify fabrication, expands design possibilities - Increases biocompatibility, and device functionality	*- Elimination of manual cutting;* *- Expanded design capabilities* *- Versatility and reproducibility* *- Improved biocompatibility* *- Design flexibility*	*- Material and resolution limitations*	[10]
Multilayer soft lithography	Collagen type I and poly-L-ornithine functionalized PDMS	- Co-culture of intestinal epithelial cells with enteric neurons to study neuro-epithelial interactions.	- Modeling complex neuro-epithelial interactions in the gut; - Co-culturing enteric neurons with intestinal epithelial cells; - Enabling neuronal innervation of the epithelial layer.	*- Physiologically relevant micro-perfusion* *- Flexible design of vascular networks* *- Compatibility with various matrices* *- Enhanced differentiation and viability* *- Drug discovery applications* *- Potential for increased biomimicry*	*- Lack of in vivo vascular features* *- Uniform distribution limiting zonation* *- Dependence on specific material properties*	[11]
Soft lithography	PDMS and gold	- Facilitating rapid, sensitive, and specific point-of-care diagnostic applications, particularly for DNA detection in clinical settings	- Complexity of integrating electrochemistry with microfluidics - Implemented Electrode functionalization difficulties functionalization techniques - Enhanced DNA immobilization - Improved sensitivity and specificity of fluid dynamics	*- Tailored microfluidic design* *- Efficient seeding and planarization* *- Visualization capabilities* *- Robust exchange of molecules* *- Maintenance of neuronal morphology* *- Limited cell cross-contamination*	*- Limited evaluation period* *- Cell contamination and infiltration* *- Lack of structural and functional assessment* *- Potential for experimental variability*	[12]
	PMMA PDMS and fibrinogen gel	- Study of vascular formation, endothelial-pericyte interactions, and immune cell behavior within a 3D ECM - Vascular disorders and analyzing inflammatory responses	- Mimicking natural vessel geometry and interactions; - Created perfusable micro-channels in 3D ECM; - Supported complex vascular cell interactions without artificial interference.	*- Functional neovessel replication* *- Endothelial barrier maintenance* *- Live confocal imaging* *- Viable and functional neovessels* *- Biological stimuli responsiveness* *- Immune cell interaction*	*- Neovessel size limitation* *- Shear stress levels* *- Hydrogel properties* *- Flow and vessel integrity*	[13]
	PDMS	- Prostate cancer disease - Study of the interactions between prostate cancer cells and the tumor microenvironment	- Provide a more accurate representation of traditional in vitro and in vivo models - Simplify the capture of tumor-stroma interactions - Enhance prediction of therapeutic outcomes	*- Physiologically relevant model* *- Microfluidic design* *- Visualization and labeling* *- Cytokine secretion analysis* *- Drug screening and development*	*- Limited immune response representation* *- Biological and molecular limitations*	[14]

Poly(ethylene glycol) diacrylate (PEGDA)-250; Diphenyl(2,4,6-trimethylbenzoyl)phosphine oxide (TPO); 2-isopropylthioxanthone (ITX); pentaerythritol tetraacrylate (PETTA); polydimethylsiloxane (PDMS); polymethylmethacrylate (PMMA)

These advancements are crucial for creating more accurate and complex models of human diseases, which mirror physiological conditions closely [1, 2, 6, 8, 13–15].

Microfluidic in vitro models offer numerous advantages over traditional culture systems, organoids, and in vivo models [16–18]. These devices provide precise control over the cellular microenvironment, enabling the recreation of complex physiological conditions such as gradients of nutrients, oxygen, and signaling molecules [19–22]. Lauren M. Delong et al. [23] reported a 3D-printed microfluidic device that was designed and fabricated to maintain the oxygen gradient across precision-cut murine intestinal slices while being capable of coupling to external neurochemical recording techniques. The gradient was sustained from outlets below, allowing access to the slice from above for detection with fast-scan cyclic voltammetry (FSCV) and carbon-fiber microelectrodes [23]. A series of 11 outlet ports were designed to lay underneath the slice, connected to channels for delivering oxygenated versus deoxygenated media. The outlet ports were shaped in an oval design, with deoxygenated media delivered to the center of the slice and oxygenated media to the outer portion, mimicking the natural oxygen distribution in the intestine [23]. Another interesting example of controlled gradients is the novel microfluidic platform called Griddient [24]. Such type of device, designed by Cristina Sanchez-de-Diego et al., aims to study immune cell extravasation, migration, and endothelial barrier permeability in vitro [24]. It allows for the creation of transient spatial and temporal gradients on demand, leveraging capillary forces to generate reconfigurable gradients in a simplistic yet robust manner. The Griddient system consisted of an array of 32 microfluidic chambers for 3D culture connected to reservoir wells, enabling the manipulation of nutrient concentration throughout the platform [23]. That study demonstrated the platform’s ability to support the migration and proliferation of natural killer (NK) cells through a collagen hydrogel, as well as the establishment of an endothelial monolayer that responds to inflammatory signals [24].

Additionally, microfluidic systems allow for the integration of multiple cell types and tissues in a controlled manner, thus facilitating the study of cell-cell interactions and tissue-level responses [22, 25, 26]. When compared to organoids, microfluidics offers superior control over spatial organization and dynamic microenvironments, allowing for a more accurate recapitulation of tissue architecture and function. While organoids represent more complex tissue structures, they often lack the precise control over cellular interactions and environmental cues that microfluidic systems provide [22, 27, 28]. However, organoids may better mimic certain aspects of tissue development and disease progression due to their three-dimensional organization and self-assembly properties [22, 29].

Microfluidic platforms also offer the possibility of high-throughput screening and automation, thus accelerating drug discovery and development processes. Furthermore, microfluidic systems have several advantages over in vivo models, including reduced cost, ethical considerations, and the ability to perform high-throughput experimentation [4, 17, 21, 22, 25, 28]. Nevertheless, they may not fully recapitulate the complexity of the in vivo environment, such as systemic interactions, immune responses, and long-term tissue remodeling [4].

Microfluidic models utilize channels to recreate the complex microarchitecture and fluid dynamics of tissues or organs, providing superior control over cellular microenvironments and enabling precise manipulation of biochemical and biophysical cues [18, 20, 22, 26, 30]. While organoids and in vivo models provide valuable insights into tissue biology and disease mechanisms, microfluidic-based models offer a complementary tool for drug screening, disease modeling, and personalized medicine, bridging the gap between traditional in vitro and in vivo approaches [16, 30, 31]. In this context, Magdalena Flont et al. [32] recently developed a new microfluidic system for creating a layered cellular cancer model with non-cancerous stroma on a poly(ethylene terephthalate) (PET) membrane. Their work aimed to mimic the complex structure of a tumor under in vitro conditions, focusing on screening anticancer drugs. The microfluidic system was designed to enable the modeling of cancer diseases in a reproducible and representative manner. By utilizing a scaffold made of a porous PET membrane, the researchers successfully demonstrated the penetration of test compounds into cancer cells through the fibroblast layer and pores in the membrane, indicating the system’s potential for 3D cell culture and testing permeability [32]. In that study, the researchers co-cultured cancer cells with non-malignant cells on the biocompatible polymer membrane to assess the toxicity of anticancer compounds. Using CAM/PI staining for each tested drug concentration, the researchers were able to evaluate the cytotoxicity and photocytotoxicity of the compounds. Additionally, the microfluidic system allowed for the analysis of the effectiveness of photodynamic therapy in treating melanoma and chemotherapy in treating breast cancer [32]. Furthermore, the developed microsystem provided a more advanced alternative to standard two-dimensional in vitro cell models by enabling cell culture in the form of a double monolayer. That arrangement facilitated the regular and reproducible organization of cells in the culture, maintaining intercellular communication essential for studying drug screening and diffusion into cancer cells. The versatility of the system, coupled with the use of porous membranes, opens up possibilities for testing membrane permeability, drug penetration, and developing 3D models of various cancer types or non-malignant tissues [32].

**Fig. 2:**
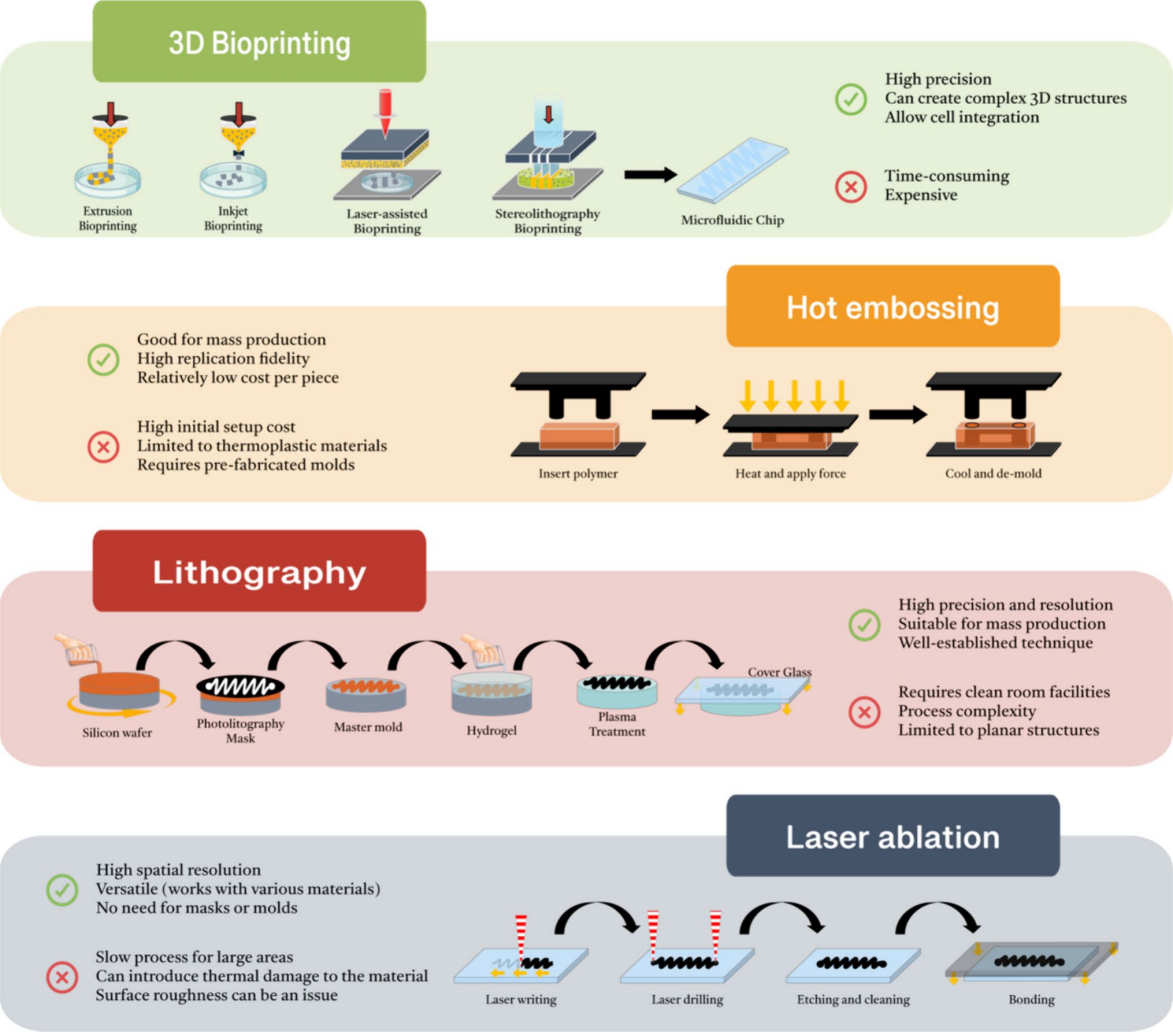
Main biofabrication techniques employed in the fabrication development of soft microfluidic devices: a highlighting illustration of the major methodologies, advantages, and limitations of 3D bioprinting, hot embossing, lithography, and laser ablation techniques

Innovations in hot embossing and imprinting, lithography, soft lithography, and emerging techniques such as 3D printing and laser ablation (Fig. 2) have markedly refined the precision of bioink deposition [5, 33]. These methodologies can facilitate the fabrication of intricate constructs that emulate the cellular milieu with unprecedented fidelity. The establishment of microphysiological systems using these technologies brings a new era, enabling the recapitulation of tissues and organ-specific features on a microscale [33–35]. By employing microfluidics, Chong Shen et al. developed a novel lung-on-a-chip platform based on a biomimetic hydrogel membrane. That platform aimed to mimic the alveolar structure by sandwiching the hydrogel membrane between two PDMS parts, with human umbilical vein endothelial cells (HUVECs) and human pulmonary alveolar epithelial cells (HPAEpiCs) seeded on each side to form the alveolar-capillary barrier. The microfluidic device allowed for cyclic membrane stretching by introducing air into the top chamber and fluid flow through the bottom chamber to manipulate shear stress on HUVECs and deliver nutrients to HPAEpiCs [36]. In the context of drug screening and disease modeling, the membrane deformation influenced fluid flow-induced shear stress on HUVECs. Computational fluid dynamics simulations were used to analyze the peak and mean shear stress on the membrane at varying flow rates and strains. The flow rate chosen aimed to approximate the shear stress on the human capillary wall. The study demonstrated that increasing strain significantly enhanced velocity and shear stress in the chamber due to the compacted chamber by the stretched hydrogel membrane [36]. Such type of innovative microfluidic lung-on-a-chip model provided a platform for studying drug sensitivity for individualized treatment of lung cancer [36]. Additionally, it offers a valuable tool for modeling pulmonary fibrosis and understanding the role of alveolar cells under mechanical stress in contributing to fibrosis [36].

The described cutting-edge approaches not only enhance resolution and control but also lead the way in personalized medicine by supporting the study of disease triggers and the testing of therapies in precisely tailored environments [37].

**Fig. 3:**
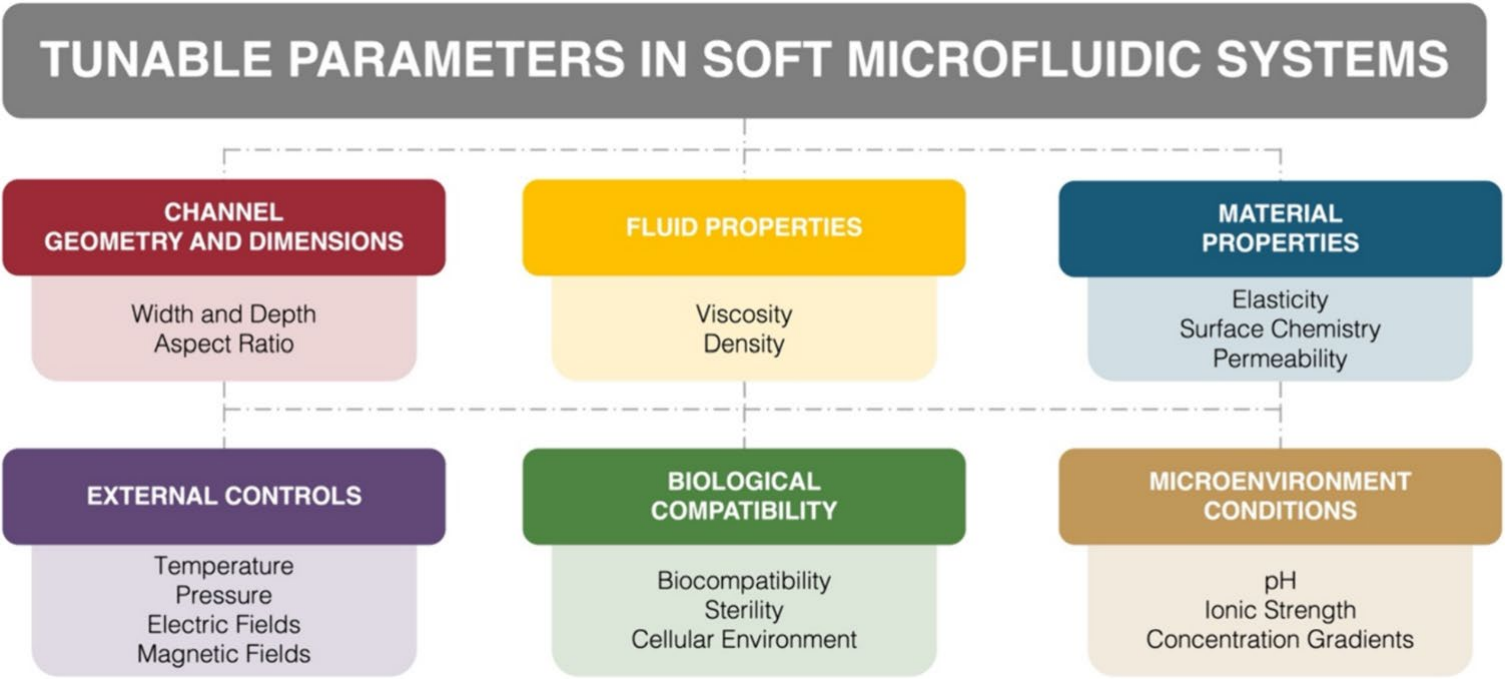
Tunable parameters in soft microfluidic systems. The key adjustable parameters are categorized into six main areas: channel geometry and dimensions, fluid properties, material properties, external controls, biological compatibility, and microenvironment conditions

**Fig. 4:**
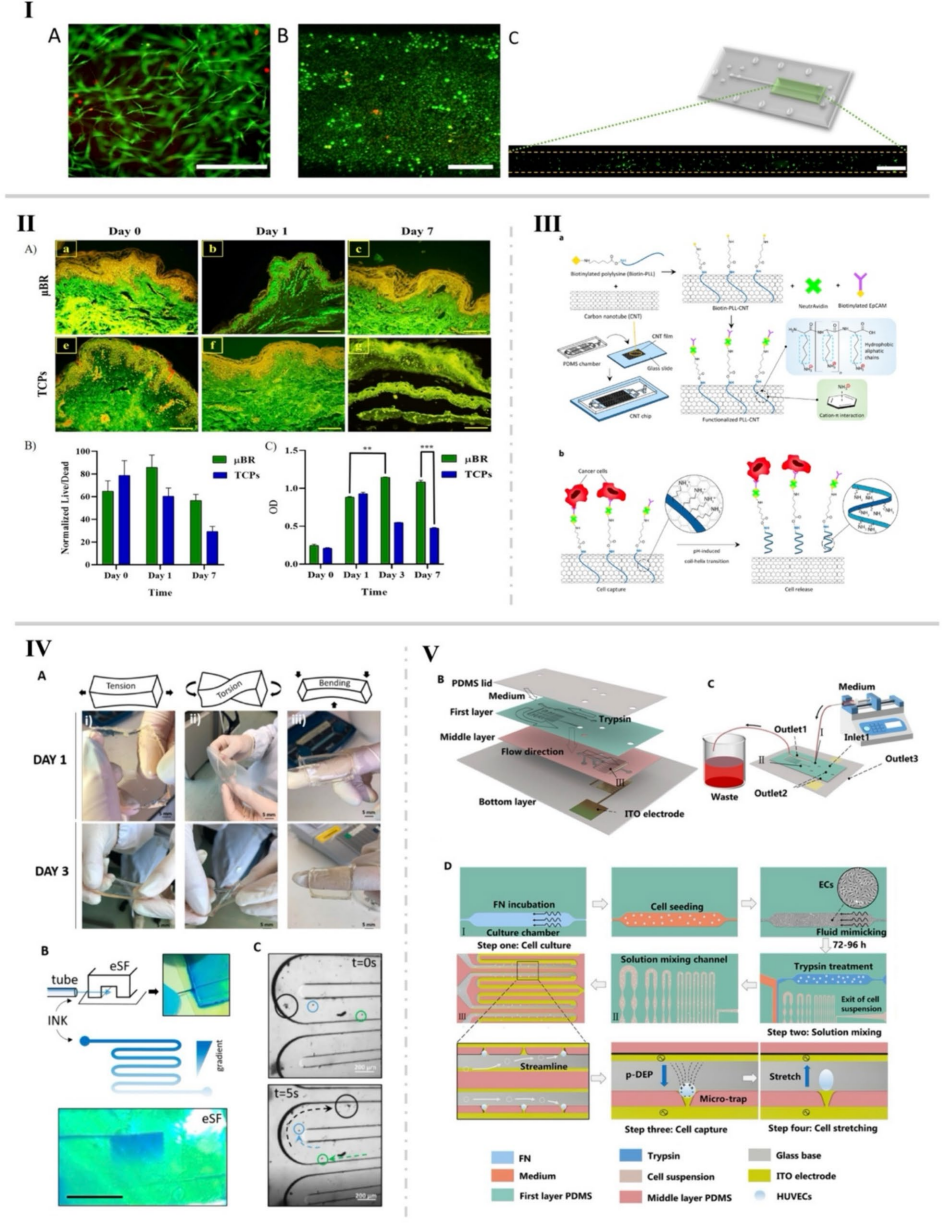
Image panel with different microfluidic devices: **I v**isualization of cell viability in microfluidic devices: (A) human fibroblasts embedded within fibrin hydrogel inside a microchannel, with green and red fluorescence indicating live and dead cells respectively. (B) human keratinocytes on fibrin hydrogel observed through confocal microscopy. (C) an overhead view of the upper chamber loaded with GFP-tagged human fibroblasts in fibrin gel, showcasing even distribution and cell spread; **II** fluorescent imaging and viability of full-thickness skin tissue models: (A) live imaging of full-thickness skin tissue in micro-bioreactors (µBR) and tissue culture plates (TCPs) on days 0, 1, and 7. (B) Graphical analysis of skin cell viability via Acridine Orange staining over 7 days. (C) Comparative viability of full-thickness skin tissues in µBR and TCP environments over 7 days, assessed by MTT assay, highlighting significant differences; **III** schematic illustration of the pH-responsive carbon nanotube (CNT) microfluidic chip: (a) the structure of the CNT chip and the process of capturing circulating tumor cells (CTCs). (b) the release process of captured CTCs under high pH conditions due to the transformation of poly-L-lysine (PLL) structure;  **IV** enzymatically crosslinked silk fibroin (eSF) hydrogel microfluidic platform: (A) the unique mechanical properties of the eSF hydrogel platform. (B) Ink perfusion experiments and formation of a gradient in the serpentine channel. (C) Microscopy snapshots of microstructure flow within the serpentine channel; **V** multi-layer integrated microfluidic chip design: (B) schematization of the chip’s structure. (C) Diagram of the chip’s architecture emphasizing oxygen and plasma bonding technology. (D) The operating principles of the microfluidic device; images adapted with permission from references [ 34, 35, 40–42 ], copyright 2021 Springer Nature, copyright 2023 Springer Nature, copyright 2022 American Chemical Society, copyright 2022 Authorea, and copyright 2023 Royal Society of Chemistry, respectively

 In 2021, Valencia et al. [35] introduced a ground-breaking microfluidic system capable of creating multi-layered tissues-on-chips. A novel approach to generate 3D multilayer tissue models on microfluidic platforms was established using a “cut and assemble” method. A parallel flow technique for bilayer tissue deposition was employed, as well as a new vinyl-based microfluidic device, to demonstrate the dynamic upkeep of multilayer tissues in conditions that simulate the function of blood vessels. Valencia’s preliminary experiments showed promise (Fig. 4I), demonstrating that this biochip improves the development and maintenance of multilayer tissues, hence increasing the potential for novel and improved biological models for complex biological interaction research [35]. In 2023, Mohamadali et al. [34] developed a cutting-edge skin-on-a-chip microfluidic device through soft lithography. A technology that realistically mimics the entire thickness of human skin (Fig. 4II), including the epidermis and dermis layers, was successfully created. That model accurately replicated the skin’s vascular system with polydimethylsiloxane (PDMS) microchannels, thus enabling efficient nutrition transportation, which is critical for skin tissue viability and mechanical integrity. The tensile strength of the skin sample in the microfluidic device decreased by about 30% after 1 week, while the mechanical strength for traditional culture platforms decreased to 70% during the same timeframe. Such type of innovation outperforms traditional culture methods in terms of skin tissue health, moisture absorption, structure, gene expression, and longevity over at least a week. Its operational simplicity and cost-effectiveness render it an invaluable tool for research and development, marking a significant leap forward from previous methods [34]. Mohamadali’s contribution has important implications, offering a more thorough and realistic model of human skin’s physiological processes [34]. This skin-on-a-chip model is expected to transform personalized medicine by allowing for more precise modeling of complex cellular interactions. It has also the potential to revolutionize drug discovery and tissue engineering, reducing reliance on animal models in favor of more relevant human tissue models. The study’s implications span medicine, pharmacology, and tissue engineering, underscoring its importance for advancing personalized medicine and boosting our understanding of tissue biology and disease pathways. To further enhance these models, we can adjust essential tunable parameters to optimize soft microfluidic systems (Fig. 3). These parameters can be meticulously tuned to enhance the performance and adaptability of soft microfluidic systems, facilitating advancements in biomedical research, chemical synthesis, and high-throughput screening applications. The combination of these tunable factors provides a versatile platform for developing innovative microfluidic solutions.

For example, the selective use of sophisticated materials in bioink formulations is critical to microfluidic platforms’ performance [7, 38, 39]. These often involve stimuli-responsive, or “smart” materials, extending the boundaries for creating small systems endowed with complex features that may enable the development of sophisticated diagnostic modalities, and targeted drug delivery systems [7, 39]. The selection of stimuli (e.g., pH, temperature, light) and type of biomaterials (e.g., methacrylated gelatin, silk fibroin, poly (dimethylacrylamide)) are intricately tied to the final purpose. Hong et al. [40] reported a novel microfluidic technology that uses a pH-responsive carbon nanotube (CNT) film to efficiently capture and release cancer cells from blood samples. That microfluidic device comprised two main components: (i) a bottom-layer glass slide coated with a CNT film functionalized with pH-responsive poly-L-lysine (PLL) connected to anti-epithelial cell adhesion molecule antibodies and (ii) a top-layer PDMS cover with herringbone channels. The setup significantly enhanced cell collision with the functionalized CNT film, thus leading to an 86.7% capture efficiency. By means of adjusting the pH to trigger a conformational shift in the PLL, the device achieved an 84.7% release efficiency, with the released cells maintaining a high level of biological viability (84.6%). That system, schematized in Fig. 4III, represented a promising approach for the capture and release of biologically viable circulating tumor cells (CTC’s) for downstream molecular and functional studies. It successfully demonstrated the strategic use of sophisticated materials and stimuli-responsive features for microfluidic platforms [40].

The development and application of bioinks in microfluidics for applications such as tissue engineering, pharmacological studies, and organ-on-a-chip frameworks necessitate a thorough assessment of their mechanical and biological properties [7, 39]. In a recent study, Mariana Carvalho et al. developed a biomimetic and soft lab-on-a-chip platform using enzymatically crosslinked silk fibroin (eSF) hydrogel for modeling colorectal tumors [41]. That innovation aimed to address the limitations of conventional microfluidic systems, which rely on solid, non-biodegradable, and non-biocompatible materials, by incorporating biological components into soft microfluidic systems [41]. The proposed approach holds the promise of revolutionizing in vitro cell and tissue culture and modeling using hydrogel-based microfluidic technologies. The innovative platform was developed using a new methodological approach, employing eSF hydrogels, known for their unique tunability and mechano-chemical capabilities, and ideal structural fidelity [41]. In that study, it was demonstrated that the higher the concentration of eSF, the higher the G’, or the stiffness (Fig. 4IV). The 14% eSF hydrogel microfluidic platform presented a G’ of 7172 ± 605 Pa. The innovative fabrication approach has led to the creation of a microfluidic device with encapsulated living cells. That device, unlike traditional PDMS-based systems that are unsuitable for encapsulating cells, can successfully mimic the dynamic 3D microenvironment of colorectal cancer and accurately measure its reaction to chemotherapy agents, and can possibly envisioning the creation of vascularized in vitro models. The platform showed outstanding structural stability and ability to perfuse fluid while showing in vivo-like biological reactions. The eSF hydrogel’s stimuli-responsiveness can allow envisioning the development of highly customizable in vitro testing platforms, with applications ranging from in vitro disease modeling to drug screening, and the broader field of tissue engineering [41]. These bioinks are tailored to allow important cellular processes, including nutrition exchange, cell viability, proliferation, and adhesion while maintaining compatibility with the microfluidic architecture [43]. Moreover, the device design is critical, with current techniques aiming to accurately mimic physiological conditions. These devices have flexible conduits and compartments that can simulate the complex blood vessel architectures and dynamic mechanical stresses that cells encounter in vivo, such as shear stress and compression [44]. The incorporation of diverse cellular populations and the establishment of gradients for essential components such as nutrients and oxygen within these constructs enable a more accurate simulation of the cellular microenvironment. Such type of design concept goes way beyond standard modeling techniques by shedding light on cellular dynamics and interactions in an environment that closely resembles in vivo conditions. Interestingly, Hao Yang et al. developed an innovative microfluidic chip that combines dynamic cell culture and dielectrophoretic manipulation for the “in situ” assessment of endothelial cell mechanics [42]. The device mimics the vascular microenvironment and allows for the study of the biomechanical effects of fluid shear stress, TNF-α, and blebbistatin on HUVECs. That multi-layer microfluidic device was created using PDMS casting and oxygen plasma bonding, enabling precise control of cell culture conditions and mechanical measuring operations (Fig. 4V). The main results revealed that increased fluid shear stress enhances Young’s modulus of HUVECs, thus indicating the importance of hemodynamics in cellular biomechanics, whereas TNF-α and blebbistatin significantly reduce HUVECs stiffness [42]. The research contribution introduced a novel vascular-mimetic dynamic culture system and monitoring approach, significantly improving the study’s efficiency and accuracy on hemodynamics and pharmacological mechanisms. The microfluidic chip’s ability to respond to changes in the environmental conditions presents an invaluable tool for cardiovascular disease research, for example, offering significant advantages in simulating in vivo conditions, high throughput analysis, and potential applications in drug screening and disease modeling [42].

## Challenges and future directions

Soft microfluidic systems have unequivocally revolutionized the landscape of disease modeling, allowing for subtle and customized investigations that were previously out of reach. These models provide unprecedented opportunities for studying disease ontogeny and therapeutic approaches within a controlled and reproducible setting. Nevertheless, challenges still remain in the advancement of biofabrication and soft microfluidic systems, despite the strides made in diagnostics and disease modeling. A critical obstacle is related to the need to replicate complex tissue structures and achieve functional vascularization within microfluidic devices. Such type of grand challenge calls for significant inventive approaches and designs for emulating the sophisticated architecture and functionalities of living systems in vitro. The refinement of endothelial cell incorporation to mimic in vivo vasculature [11] represents a stride towards this goal. Moreover, the assessment of neuro-epithelial contacts within co-cultures, aimed at unraveling the complexities of gastrointestinal diseases [12], underscores the need for enhanced biological mimicry.

Additionally, ensuring the scalability and reproducibility of these systems for widespread application in diagnostics and disease modeling is of paramount importance. Addressing these technical challenges is imperative to unlock the full potential of soft microfluidics, ultimately enabling more effective tailored cost-effective and personalized solutions for healthcare. In vitro testing is vital for validating microfluidic chips [21, 24, 32, 36]. This includes assessing fluid dynamics and mixing efficiency to ensure accurate replication of physiological fluid flows [45, 46]. In vitro cytotoxicity screening and live/dead cell staining can help assessing that materials are non-toxic to cells. Simulating biological conditions, such as controlled temperature and pH, best emulates human physiological settings for accurate biological interaction testing [45–48]. By means of applying such types of methods, researchers ensure that new devices are precise, reliable, and biocompatible, demonstrating improvements over existing technologies and advancing personalized healthcare solutions in diagnostics and disease modeling. Advancements in biosensor platforms are also anticipated, aiming for broader clinical applications including comprehensive disease diagnostics, therapeutics, and potentially real-time biomolecular interaction monitoring in point-of-care settings [49].

Looking ahead, the future of biofabrication and soft microfluidic systems holds great innovation potential. As research progresses, a key focus will be refining system precision and functionality, ensuring that they not only mimic but also replicate the dynamic mechanical properties of living tissues more closely. A particular area of interest lies in the versatility of these systems to encapsulate a variety of cell types fostering the creation of more intricate and mimetic cellular arrangements and microenvironments. Future research aims to refine the precision and functionality of these systems as there is a marked interest in integrating more complex cellular and microbial components to faithfully replicate organ physiology, particularly within the gastrointestinal tract for extensive pharmacokinetic analysis [50].

The integration of Clustered Regularly Interspaced Short Palindromic Repeats (CRISPR) within microfluidic devices is another promising venture. Such type of synergistic approach could yield unprecedented advancements in the field. By means of combining the unparalleled specificity of CRISPR technologies with the versatility and efficiency of microfluidic systems, scientists are on the edge of developing highly targeted therapies for genetic disorders, enabling the correction of mutations at their source with minimal off-target effects [51, 52]. Furthermore, this integration paves the way for high-throughput screening of genetic interactions and drug responses in real-time, offering insights into complex biological processes at an unparalleled scale. The ability to manipulate genes within microenvironments that closely mimic the physiological conditions of living tissues could revolutionize our approach to understanding disease mechanisms, leading to the development of novel treatments and diagnostics [51, 52].

Addressing these challenges and harnessing the innovation potential of biomaterials, biofabrication methods and soft microfluidic systems will be instrumental in decoding complex biological interactions and pathologies, contributing significantly to the evolution of personalized medicine and revolutionizing the landscape of disease modeling and treatment.

## Conclusion

The field of biofabrication has undergone remarkable strides in recent years, marking a transformative *era* in diagnostics and disease modeling propelled by the advent of soft microfluidic systems. Innovations in fabrication techniques such as 3D printing, soft lithography, and laser ablation have facilitated the creation of intricate, three-dimensional in vitro models that can better emulate human tissues and organs with unprecedented fidelity. These advancements not only enhance disease modeling accuracy but also lay the foundation for personalized medicine, offering tailored therapeutic strategies based on individual physiological responses. The groundbreaking work of several researchers and pioneers underscores the transformative potential of soft microfluidic systems in creating multi-layered tissue models and skin-on-a-chip devices, enabling in-depth studies of complex biological interactions and drug responses within controlled microenvironments. Moreover, the strategic use of advanced soft biomaterials and stimuli-responsive bioinks can open up new avenues for the development of sophisticated diagnostic tools and targeted drug delivery systems. As biofabricated microfluidic systems expand into clinical settings, they present an exciting frontier for personalized diagnostics, patient-specific treatment planning and stratification, and even the fabrication of bespoke tissue implants for regenerative medicine. The journey from conceptual frameworks to practical applications epitomizes interdisciplinary collaboration, drawing upon expertise from materials science, biology, engineering, and computer science. With continued innovation, these systems promise to revolutionize medical approaches, profoundly impacting healthcare outcomes worldwide.

## Data Availability

Research data are not shared.
